# A Provably Secure Revocable ID-Based Authenticated Group Key Exchange Protocol with Identifying Malicious Participants

**DOI:** 10.1155/2014/367264

**Published:** 2014-06-01

**Authors:** Tsu-Yang Wu, Tung-Tso Tsai, Yuh-Min Tseng

**Affiliations:** ^1^Shenzhen Graduate School, Harbin Institute of Technology, Shenzhen 518055, China; ^2^Shenzhen Key Laboratory of Internet Information Collaboration, Shenzhen 518055, China; ^3^Department of Mathematics, National Changhua University of Education, Jin-De Campus, Changhua City 500, Taiwan

## Abstract

The existence of malicious participants is a major threat for authenticated group key exchange (AGKE) protocols. Typically, there are two detecting ways (passive and active) to resist malicious participants in AGKE protocols. In 2012, the revocable identity- (ID-) based public key system (R-IDPKS) was proposed to solve the revocation problem in the ID-based public key system (IDPKS). Afterwards, based on the R-IDPKS, Wu et al. proposed a revocable ID-based AGKE (RID-AGKE) protocol, which adopted a passive detecting way to resist malicious participants. However, it needs three rounds and cannot identify malicious participants. In this paper, we fuse a noninteractive confirmed computation technique to propose the first two-round RID-AGKE protocol with identifying malicious participants, which is an active detecting way. We demonstrate that our protocol is a provably secure AGKE protocol with forward secrecy and can identify malicious participants. When compared with the recently proposed ID/RID-AGKE protocols, our protocol possesses better performance and more robust security properties.

## 1. Introduction

In the past, group-oriented applications, such as collaboration works and teleconference, were popularly and widely used in the Internet. Authenticated group key exchange (AGKE) protocol [1] is a cryptographic primitive which provides secure group communications for users in cooperative and distributed applications. During executing the protocol, group participants not only cooperatively generate a common key which is used to encrypt the transmitted messages but also authenticate the participants' identities.

The existence of malicious participants is a major threat for AGKE protocols. The goal of malicious participants is to disturb the establishing of common keys. Hence, how to resist malicious participants in AGKE protocols becomes a critical research. Typically, there are two detecting ways to resist malicious participants. (I) Passive detection [2–4]: it involves an explicit key confirmation approach in AGKE protocols. The resulted protocols only detect the existence of malicious participants and an additional round is required. (II) Active detection [5, 6]: it adopts a noninteractive confirmed computation technique into AGKE protocols. The resulted protocols can identify the identities of malicious participants without additional round. However, the computational cost of active detection is time-consuming than the one of passive detection.

Quite recently, the revocable identity- (ID-) based public key system (R-IDPKS) was proposed to solve the revocation problem of users in the ID-based public key system (IDPKS). The concept of IDPKS was introduced by Shamir [7] in 1984 and was practiced by Bonch and Franklin [8] in 2001. Indeed, they [8] had suggested a solution that the private key generator (PKG) renews these nonrevoked users' private keys periodically to answer the revocation problem in the IDPKS. The approach can be used to revoke the compromised or misbehaving users. Nevertheless, the heavy workload arose from the PKG for renewing users' private keys periodically.

In 2008, Boldyreva et al. [9] proposed a revocable ID-based encryption (RIBE) scheme by using binary tree. Their scheme can reduce the PKG's workload mentioned in the Boneh-Franklin solution [8]. However, this scheme is based on a weak security model called the relaxed selective-ID model [10]. In 2009, Libert and Vergnaud [11] relied on Boldyreva et al.'s RIBE to present a secure RIBE scheme under an adaptive-ID model. Recently, Seo and Emura [12] demonstrated Boldyreva et al.'s scheme [9] is vulnerable to decryption key exposure and then proposed a provably secure tree based RIBE scheme. Subsequently, Seo and Emura [13] presented a hierarchical RIBE scheme to solve the open problem mentioned in [11].

In 2011, Tseng and Tsai [14] proposed a practical RIBE scheme over a public channel. The key construction of the Tseng-Tsai scheme is different from the previous schemes [9, 11–13]. In [14], each user's private key consists of a fixed initial private key and an update key, where the update key is renewed along with the current period. For an honest (nonrevoked) user, the PKG periodically issues new update key and sends it to the user via a public channel. Upon receiving the new updating key, the user can renew her/his private key by herself/himself. To revoke a malicious user, the PKG only stops issuing the new update key in current period. Thus, the user cannot compute the newest private key. In other words, she/he cannot execute any cryptographic behaviors in later periods. Later on, several revocable ID-based cryptographic schemes based on the Tseng-Tsai R-IDPKS [14] were presented such as encryption [15], signature [16, 17], authenticated group key exchange (AGKE) [4], and signcryption [18].

In 2012, Wu et al. [4] proposed the first provably secure revocable ID-based AGKE (RID-AGKE) protocol. Their protocol adopted a passive detecting way to resist malicious participants. However, it requires three rounds and cannot identify the identities of malicious participants. In this paper, we fuse the key construction of Tseng-Tsai R-IDPKS [14] and a noninteractive confirmed computation technique [6] to present a two-round RID-AGKE protocol with identifying malicious participants. In our protocol, each group participant can confirm whether the broadcast values are correctly computed by other participants. Based on the detecting approach, our protocol can easily identify the participants who maliciously broadcast the incorrect values to disturb the common key establishing. The framework and security notions for RID-AGKE protocols are defined to formalize possible threats and attacks. We demonstrate the security of our protocol in the random oracle model [19] and under two mathematical assumptions (the computational Diffie-Hellman and the decisional bilinear Diffie-Hellman). Finally, we make the comparisons between our protocol and the recently proposed ID/RID-AGKE protocols to show the advantages of the proposed protocol.

The rest of this paper is organized as follows. We briefly review the concepts of bilinear pairings and related mathematical problems in Section 2. The security model and notions of RID-AGKE are presented in Section 3. We propose a concrete RID-AGKE protocol in Section 4. Security analysis of the proposed RID-AGKE protocol is demonstrated in Section 5. We make the performance analysis and comparisons in Section 6. Conclusions are drawn in Section 7.

## 2. Preliminaries

In this section, we briefly review the properties of bilinear pairings and related mathematical problems. For the details, a reader can refer to [8, 20, 21] for full descriptions.

### 2.1. Bilinear Pairings

Let *G*_1_ and *G*_2_ be two groups of a large prime order *q*, where *G*_1_ is an additive cyclic group and *G*_2_ is a multiplicative cyclic group. A bilinear pairing *e* is a map defined by *e* : *G*_1_ × *G*_1_ → *G*_2_ and satisfies the following three conditions.

1. *Bilinearity*: for all *P*, *Q* ∈ *G*_1_ and *a*, *b* ∈ *Z*_*q*_, *e*(*aP*, *bQ*) = *e*(*P*,*Q*)^*ab*^.
2. *Nondegeneracy*: there exist *P*, *Q* ∈ *G*_1_ such that *e*(*P*, *Q*) ≠ 1.
3. *Computability*: for all *P*, *Q* ∈ *G*_1_, there exists an algorithm to compute *e*(*P*, *Q*).

### 2.2. Mathematical Hard Problems and Assumptions

Here, we present two mathematical hard problems and define the corresponding assumptions as follows.

1. *Computational Diffie-Hellman (CDH) problem*: given *P*, *aP*, *bP* ∈ *G*_1_ for some *a*, *b* ∈ *Z*_q_*, the CDH problem is to compute *ab* *P* ∈ *G*_1_.
2. *Decisional bilinear Diffie-Hellman (DBDH) problem*: given *P*, *aP*, *bP*, *cP*, *dP* ∈ *G*_1_ for some *a*, *b*, *c*, *d* ∈ *Z*_q_*, the DBDH problem is to distinguish (*P*, *aP*, *bP*, *cP*, *dP*, *e*(*P*,*P*)^*abc*^ from (*P*, *aP*, *bP*, *cP*, *dP*, *e*(*P*,*P*)^*d*^).

**Definition 1 (CDH assumption)**

Given *P*, *aP*, *bP* ∈ *G*_1_ for some *a*, *b*∈_*R*_ *Z*_q_*, there does not exist a probabilistic polynomial-time algorithm *A* with a nonnegligible probability to compute *ab* *P* ∈ *G*_1_. The advantage of *A* within running time *t* is defined as *Adv*_*CDH*_(*t*) = Pr[*A*(*P*, *aP*, *bP*) = *ab* *P* | *P*, *aP*, *bP* ∈ *G*_1_].

**Definition 2 (DBDH assumption)**

Given *P*, *aP*, *bP*, *cP*, *dP* ∈ *G*_1_ for some *a*, *b*, *c*, *d*∈_*R*_ *Z*_q_*, there does not exist a probabilistic polynomial-time algorithm *A* with nonnegligible probability to distinguish (*P*, *aP*, *bP*, *cP*, *dP*, *e*(*P*,*P*)^*abc*^) from (*P*, *aP*, *bP*, *cP*, *dP*, *e*(*P*,*P*)^*d*^). The advantage of *A* within running time *t* is defined as *Adv*_*DBDH*_(*t*) = Pr[*A*(*e*(*P*,*P*)^*abc*^, *e*(*P*,*P*)^*d*^) = 1 | *P*, *aP*, *bP*, *cP*, *dP* ∈ *G*_1_].

## 3. Model and Notions

In this section, we define the model and notions for RID-AGKE protocol. Note that some of the following definitions and notations are referred to in [4, 6, 22–24].

*Initialization.* The initialization of RID-AGKE protocol has three algorithms.

(1)* Setup Algorithm.* This algorithm is a probabilistic algorithm which takes as input a security parameter *k* and a total number *z* of periods. It returns a system private key *s* and public parameters* param*. Note that the whole life time of the system is divide into distinct periods 1, 2,…, *z*. Here,* param* is made public.

(2)* Initial Key Extract Algorithm.* This algorithm is a deterministic algorithm which takes as input the system private key *s* and a participant's identity *ID*. It returns the participant's initial private key *D* *ID*.

(3)* Key Update Algorithm.* This algorithm is a deterministic algorithm which takes as input the system private key *s*, a participant's identity *ID*, and a period index *j*, where 1 ≤ *j* ≤ *z*. It returns the participant's update key *T* *ID*_*j*_.

Here, note that the participant's private key for period *j* is defined by *D* *ID*_*j*_ = *D* *ID* + *T* *ID*_*j*_.

*Related Notions.* For simplicity, there is a fixed set *G* = {*U*_1_, *U*_2_,…, *U*_*n*_} with polynomial size of potential participants. Assume that each participant *U*_*i*_ has a unique identity *ID*_*i*_ ∈ {0,1}*. Any subset of *G* may run a RID-AGKE protocol many times (possibly concurrently) in some period index *j* to establish a group session key, where 1 ≤ *j* ≤ *z* and *z* is a total number of periods. Note that the set of participants' identities, **I** **D** = {*ID*_1_, *ID*_2_,…, *ID*_*n*_} is known by all participants (including adversary).

An instance *t* of participant *U* in period *j* is denoted by Π_*U*_^*j*(*t*)^, where *t* is a positive integer. Each instance Π_*U*_^*j*(*t*)^ has associated with seven variables as follows.

- *state*_*U*_^*j*(*t*)^: it presents the current state of instance Π_*U*_^*j*(*t*)^.
- *acc*_*U*_^*j*(*t*)^ and *term*_*U*_^*j*(*t*)^: they take Boolean values to demonstrate whether Π_*U*_^*j*(*t*)^ *has accepted* or terminated. Informally, we say that an instance* has accepted* meaning that it does not detect any incorrect behavior. An instance is called terminating if it has sent and received messages. Note that a terminated instance may also possibly accept.
- *used*_*U*_^*j*(*t*)^: it indicates whether Π_*U*_^*j*(*t*)^ is used in a RID-AGKE protocol.
- *pid*_*U*_^*j*(*t*)^: the partner ID of instance Π_*U*_^*j*(*t*)^ is a set which contains the identities of participants in the group with whom Π_*U*_^*j*(*t*)^ wants to establish a group session key (including *U* itself).
- *sid*_*U*_^*j*(*t*)^: the session ID of instance Π_*U*_^*j*(*t*)^ is a concatenation of all messages sent and received by the instance in a given execution of RID-AGKE protocol.
- *sk*_*U*_^*j*(*t*)^: a group session key which is accepted by instance Π_*U*_^*j*(*t*)^.

In the following definitions, we will only focus on the three variables *pid*_*U*_^*j*(*t*)^, *sid*_*U*_^*j*(*t*)^, and *sk*_*U*_^*j*(*t*)^. The remaining variables will be left implicit. We say that two instances Π_*U*_^*j*(*t*)^ and Π_*U*′_^*j*(*v*)^ are* partnered* if (1) they have accepted the same group session key; (2) *sid*_*U*_^*j*(*t*)^ = *sid*_*U*′_^*j*(*v*)^; and (3) *pid*_*U*_^*j*(*t*)^ = *pid*_*U*′_^*j*(*v*)^.

*Adversarial Model.* An adversary *A* can be viewed as a probabilistic polynomial-time algorithm. Here, we assume that *A* can potentially control all communications in a RID-AGKE protocol. The interaction between *A* and instances of participants in the protocol is modeled by the following oracles.

- *Execute *(*V*, *j*): when *A* makes* Execute query* on (*V*, *j*), it executes the RID-AGKE protocol between the unused instances of participants in *V* for period index *j* and then returns a transcript of the execution, where *V* is a subset of *G*. Here,* Execute query* is used to model passive attacks.
- *Inextract *(*ID*_*U*_): when *A* makes* Initial key extract query* on identity *ID*_*U*_, it generates an initial private key *D* *ID*_*U*_ corresponding to *ID*_*U*_ and returns it to *A*, where *ID*_*U*_ ∉ **I** **D**.
- *Kupdate *(*ID*_*U*_, *j*): when *A* makes* Key update query* on (*ID*_*U*_, *j*), it generates an update key *T* *ID*_*U*,*j*_ corresponding to (*ID*_*U*_, *j*) and returns it to *A*, where *ID*_*U*_ ∉ **I** **D** and *j* is a period index.
- *Send *(*U*, *j*, *t*, *M*): when *A* makes* Send query* on (*U*, *j*, *t*, *M*), it sends message *M* to instance Π_*U*_^*j*(*t*)^ and then returns the reply generated by this instance according to procedures of RID-AGKE protocol.
- *Reveal *(*U*, *j*, *t*): when *A* makes* Reveal query* on (*U*, *j*, *t*), it returns a group session key *sk*_*U*_^*j*(*t*)^ for a terminated instance Π_*U*_^*j*(*t*)^. Here,* Reveal query* is used to model known session key attacks.
- *Corrupt *(*ID*_*U*_, *j*): when *A* makes* Corrupt query* on (*ID*_*U*_, *j*), it returns a private key *D* *ID*_*U*,*j*_ of *ID*_*U*_ in period *j*. Note that* Corrupt query* models the corruption of this participant at a time in which it is not currently executing the protocol. We say that a participant *U* is honest if and only if no* Corrupt query* has been made by *A*.
- *Test *(*U*, *j*, *t*, ): at any time, the adversary *A* makes* Test query* only once to this oracle during *A*'s execution. In this moment, a random coin *b* ∈ {0,1} is selected. If *b* = 1, a group session key *sk*_*U*_^*j*(*t*)^ is retuned. Otherwise, a random value is retuned. Here,* Test query* is used to model the semantic security of group session key.

According to the above adversarial model, we define two types of adversaries. A* passive adversary* is allowed to make* Execute*,* Reveal*,* Corrupt*, and* Test* queries. An* active adversary* is allowed to make the above all queries. In order to get more precise analysis, we still use* Execute query* though it can be substituted by making* Send query* repeatedly.

**Remark 3**

According to the adversarial model above, the adversary *A* can compute the participant *U*'s private key *D* *ID*_*U*,*j*_ = *D* *ID*_*U*_ + *T* *ID*_*U*,*j*_ for period index *j* while *A* makes both* Initial key extract query* on *ID*_*U*_ and* Key update query* on (*ID*_*U*_, *j*) simultaneously. Hence, we disallow *A* to make both queries in the same time.

*Correctness. A* RID-AGKE protocol is called* correct* if the following three conditions hold.

1. All participants are honest and all messages are delivered honestly.
2. *acc*_*U*_^*j*(*t*)^ = *acc*_*U*′_^*j*(*v*)^ = “True” and *sk*_*U*_^*j*(*t*)^ = *sk*_*U*′_^*j*(*v*)^.
3. *sid*_*U*_^*j*(*t*)^ = *sid*_*U*′_^*j*(*v*)^and *pid*_*U*_^*j*(*t*)^ = *pid*_*U*′_^*j*(*v*)^ for all participants *U*, *U'* ∈ *V*⊆*G* with instances Π_*U*_^*j*(*t*)^ and Π_*U*′_^*j*(*v*)^.

*Freshness.* We say that an instance Π_*U*_^*j*(*t*)^is called* fresh* (or called holding a* fresh* group session key *sk*_*U*_^*j*(*t*)^) if the following three conditions hold.

1. Π_*U*_^*j*(*t*)^ has accepted a group session key *sk*_*U*_^*j*(*t*)^.
2. Neither Π_*U*_^*j*(*t*)^ nor its partners have been made* Reveal query*.
3. No* Corrupt query* has been made on *ID*_*V*_ ∈ *pid*_*U*_^*j*(*t*)^ before* Send query* to Π_*U*_^*j*(*t*)^ or* Send query* to Π_*U'*_^*j*(*v*)^, where *ID*_*U'*_ ∈ *pid*_*U*_^*j*(*t*)^.

Here, we assume all instances are* fresh*. Note that the notion of* freshness* is defined appropriately for the purpose of forward secrecy.

*Secure RID-AGKE.* A secure RID-AGKE protocol contains the following four parts.

(1)* Freshness.*

(2)* Security of RID-AGKE Protocol*. The security of RID-AGKE protocol is defined in the following game played between an active adversary *A* and a set of instances:

- *initialization*: the system private key, public parameters, and participants' private keys are generated in this phase;
- *query*: A may make different types of queries to oracles and gets back the answers corresponding to the RID-AGKE protocol;
- *guess*: finally, the adversary *A* outputs its guess for the coin *b* in* Test query* and terminates.

In this game, the goal of *A* is to distinguish a group session key from a random value. Let* Succ* be the event that *A* correctly guesses the coin *b* in* Test query*. The advantage of *A* in attacking a RID-AGKE protocol Ψ is defined by *Adv*_*A*,Ψ_(*k*) = |2 · Pr[*Succ*] − 1|. We say that the protocol Ψ is secure, if the advantage *Adv*_*A*,Ψ_(*k*) is negligible.

(3)* Forward Secrecy*. We say that a RID-AGKE protocol Ψ provides* forward secrecy*. It means that though an adversary *A* obtains participants' private keys in Ψ, the previous establishing group session keys is preserved. The advantage of *A* in attacking the protocol Ψ within running time *t* is defined by *Adv*_Ψ_^*RIDAGKE*-*fs*^(*t*, *q*_*ex*_, *q*_*s*_), where *q*_*ex*_ and *q*_*s*_ are the maximum numbers of making* Execute* and* Send queries*, respectively.

(4)* Authentication*. We say that a RID-AGKE protocol Ψ provides* implicit key authentication* if all participants in Ψ are guaranteed that nobody other than their partners can learn the session key. In other words, any adversary should not learn the key. Note that this security property does not guarantee that the partners have computed the key.

*Malicious Participant.* A participant *U*_*m*_ is called* malicious* in a RID-AGKE protocol Ψ if he is a legal participant but is fully controlled by adversary. The goal of malicious participant is to disturb the group key establishing in Ψ.

## 4. Concrete Protocol

In this section, we propose a concrete RID-AGKE protocol with identifying malicious participants. Our protocol fuses the Tseng-Tsai R-IDPKS [14] and a noninteractive confirmed computation technique [6]. In the initialization phase, given a security parameter *k* and a total number *z* of periods, a private key generator (PKG) executes* Setup algorithm* to generate the system private key *s* and the public parameters *param* = {*G*_1_, *G*_2_, *e*, *q*, *P*, *P*_*pub*_, *H*_1_, *H*_2_, *H*_3_, *H*_4_} defined in Notations section at the end of the paper.

When a participant *U* with identity *ID*_*U*_ ∈ {0,1}* wants to obtain her/his initial private key *D* *ID*_*U*_, the PKG runs* Initial key extract algorithm *to compute *D* *ID*_*U*_ = *s* · *H*_1_(*ID*_*U*_) = *s* · *Q* *ID*_*U*_ and returns it to *U* via a secure channel. For a nonrevoked participant *U* with identity *ID*_*U*_ in time period *j*, the PKG runs* Key update algorithm* to compute her/his update key *T* *ID*_*U*,*j*_ = *s* · *H*_2_(*ID*_*U*,_ *j*) = *s* · *R* *ID*_*U*,*j*_ and returns it to *U* via a public channel, where 1 ≤ *j* ≤ *z*. Hence, any nonrevoked participant *U* can update her/his private key *D* *ID*_*U*,*j*_ = *D* *ID*_*U*_ + *T* *ID*_*U*,*j*_ by itself in period *j*.

Let *G* = {*U*_1_, *U*_2_,…, *U*_*n*_} be a set of participants who want to establish a group session key *SK*_*j*_ in period *j*. We assume that each *U*_*i*_ has a unique identity *ID*_*i*_ ∈ {0,1}* as public key and *U*_*i*_'s private key is *D* *ID*_*i*,*j*_ = *D* *ID*_*i*_ + *T* *ID*_*i*,*j*_ for period *j*. Note that the indices are subject to modulo *n*; that is, *U*_*n*+1_ and *U*_0_ denote *U*_1_ and *U*_*n*_, respectively. Finally, *m* ∈ {0,1}* is a preknown common message by all participants. The details of proposed RID-AGKE protocol are described as follows.

*Round 1.* Each participant *U*_*i*_ randomly selects a secret value *a*_*i*_ ∈ *Z*_q_* and computes *P*_*i*_ = *a*_*i*_ · *P*, *h*_*i*_ = *H*_3_(*ID*_*i*_, *P* *ID*_*j*_, *j*, *m*, *P*_*i*_), and *V*_*i*_ = *D* *ID*_*i*,*j*_ + *a*_*i*_ · *h*_*i*_ · *P*_*pub*_, where *P* *ID*_*j*_ denotes the concatenation of all participants' identities in period *j*; that is, *P* *ID*_*j*_ = *ID*_1_||*ID*_2_||⋯||*ID*_*n*_. Finally, each *U*_*i*_ broadcasts (*ID*_*i*_, *j*, *P*_*i*_, *V*_*i*_) to other participants.

*Round 2.* Upon receiving (*ID*_*i*−1_,   *j*, *P*_*i*−1_, *V*_*i*−1_) and (*ID*_*i*+1_, *j*, *P*_*i*+1_,   *V*_*i*+1_), each *U*_*i*_ first verifies them by checking

(1) e(P,∑k∈{−1,1}Vi+k)  =?  e(Ppub,∑k∈{−1,1}H1(IDi+k)+H2(IDi+k,j)+  hi+k·Pi+k),

where *h*_*i*+*k*_ = *H*_3_(*ID*_*i*+*k*_, *P* *ID*_*j*_, *j*, *m*, *P*_*i*+*k*_). If the verification is true, each *U*_*i*_ uses her/his secret value *a*_*i*_ to compute *D*_*i*_ = *e*(*a*_*i*_ · (*P*_*i*+1_ − *P*_*i*−1_), *P*_*pub*_). Then, *U*_*i*_ randomly selects a value *r*_*i*_ ∈ *Z*_q_* and computes a tuple (*ID*_*i*_, *j*, *D*_*i*_, *α*_*i*_, *β*_*i*_, *γ*_*i*_), where *α*_*i*_ = *r*_*i*_ · *P*, *β*_*i*_ = *r*_*i*_ · (*P*_*i*+1_ − *P*_*i*−1_), *γ*_*i*_ = *r*_*i*_ · *P*_*i*_ + *w*_*i*_ · *a*_*i*_ · *P*_*pub*_, *w*_*i*_ = *H*_4_(*ID*_*i*_||*P* *ID*_*j*_||*j*||*D*_*i*_||*S*_*j*_, *P*_*i*+1_ − *P*_*i*−1_, *α*_*i*_, *β*_*i*_), and *S*_*j*_ = *P*_1_||*P*_2_||⋯||*P*_*n*_. Finally, *U*_*i*_ sends this tuple to all other participants.

*Group Key Computation.* Upon receiving all (*ID*_*k*_, *j*, *D*_*k*_, *α*_*k*_, *β*_*k*_, *γ*_*k*_) for *k* = 1, 2,…, *n* except *i*, each *U*_*i*_ verifies them by checking

(2) $$\begin{array}{c} e\left(P,\overset{n}{\underset{k=1,k\neq i}{\sum }}\gamma_{k}\right)=\overset{n}{\underset{k=1,k\neq i}{\prod }}e\left(P_{k},\alpha_{k}+w_{k}\cdot P_{pub}\right),\\ e\left(P_{k+1}-P_{k-1},\gamma_{k}\right)\overset{?}{=}e\left(\beta_{k},P_{k}\right)\cdot D_{k}^{w_{k}}. \end{array}$$

If the two verifications hold, *U*_*i*_ can confirm that each *D*_*k*_ is computed by *U*_*k*_ using her/his secret *a*_*k*_ honestly for *k* = 1, 2,…, *n* except *i*. Finally, in period *j*, each participant *U*_*i*_ can compute the group session key *SK*_*j*_ = *e*(*a*_*i*_·*P*_*i*−1_,*P*_*pub*_)^*n*^ · *D*_*i*_^*n*−1^ · *D*_*i*+1_^*n*−2^ ⋯ *D*_*i*−2_.

*Identifying Malicious Participant.* When a malicious participant *U*_*m*_ tries to send a wrong tuple (*ID*_*m*_, *D*_*m*_, *j*, *α*_*m*_, *β*_*m*_, *γ*_*m*_) to disrupt the establishment of group session key, he will be identified as a malicious participant by using the following two verifying equations: $e(P,\gamma_{k})\overset{?}{=}e(P_{k},\alpha_{k}+w_{k}\cdot P_{pub})$ and $e(P_{k+1}-P_{k-1},\gamma_{k})\overset{?}{=}e(\beta_{k},P_{k})\cdot D_{k}^{w_{k}}$. Later on, *U*_*m*_ will be deleted from the participant set *G* and other honest participants may rerun the protocol.

## 5. Security Analysis

In this section, we prove the security of the proposed RID-AGKE protocol in the random oracle model [19] and under the CDH and DBDH assumptions.

*ID and Forgery Attacks*

**Theorem 4**

The proposed RID-AGKE protocol is secure against ID and forgery attacks.

**Proof**

Note that we adopt a revocable ID-based signature (RIDS) scheme [16] in Round 1 and a pairing-based signature scheme [6] in Round 2, respectively. The two signature schemes had been proven secure against ID and forgery attacks for single signature and multiple signatures with batch verification. Therefore, the proposed RID-AGKE protocol Ψ is secure against ID and forgery attacks.

*Secure RID-AGKE Providing Forward Secrecy.* Now, we demonstrate that the proposed RID-AGKE protocol Ψ is a secure RID-AGKE providing forward secrecy. Note that we use a similar technique in [3, 4, 6] to prove Theorem 5.

**Theorem 5**

Assume that four hash functions *H*_1_, *H*_2_, *H*_3_, and *H*_4_ are random oracles. Then, the proposed RID-AGKE protocol Ψ is a secure RID-AGKE providing forward secrecy under the decisional bilinear Diffie-Hellman (DBDH) and the computational Diffie-Hellman (CDH) assumptions. Concretely,

(3) AdvΨRIDAGKE-fs(t,qex,qs)  ≤  2nqex·AdvDBDH(t)  +AdvΨforge(t),

where *q*_*ex*_ and *q*_*s*_ are total numbers of making Execute and Send queries, respectively. Note that *Adv*_Ψ_^*forge*^(*t*) denotes the advantage of any forgers successfully attacking the protocol Ψ.

**Proof**

Assume that *A* is an active adversary in attacking the proposed RID-AGKE protocol Ψ with a nonnegligible advantage. Now, we consider the two possible cases. The first case is that *A* with the advantage can impersonate a participant (i.e., forging authentication transcripts). Another case is that *A* with the advantage can break the protocol Ψ without modifying any transcripts.

*Case  1*. We assume that the adversary *A* with an adaptive impersonation ability can break the RID-AGKE protocol Ψ. Using *A*, we would like to construct a forger *F* which can return valid signature tuples (*ID*, *j*, *aP*, *V*) and (*ID*, *j*, *D*, *α*, *β*, *γ*) with respect to the proposed protocol Ψ as follows. The forger *F* first generates all needed system parameters and keys. Then, *F* simulates the oracle queries made by *A*. This simulation is called perfect indistinguishable from *A*'s oracle queries except that *A* makes* Corrupt query* on (*ID*, *j*), where *j* is a period index. If it occurs, *F* fails and stops. Otherwise, when *A* generates two signature tuples (*ID*, *j*, *aP*, *V*) and (*ID*, *j*, *D*, *α*, *β*, *γ*), *F* returns the tuples (*ID*, *j*, *aP*, *V*) and (*ID*, *j*, *D*, *α*, *β*, *γ*). Let* Forge* be the event that the adversary *A* successfully generates two valid signature tuples. Then, the probability that *F* successfully returns two valid signature tuples is bounded by Pr_*A*_[*Forge*] ≤ *Adv*_*F*,Ψ_^*forge*^(*t*) ≤ *Adv*_Ψ_^*forge*^(*t*).

*Case  2*. We assume that the adversary *A* can break the proposed RID-AGKE protocol without modifying any transcripts. We first focus on the case that *A* makes* Execute query* once on (*ID*_1_, *ID*_2_,…, *ID*_*n*_, *j*) and then extends this to the case that *A* makes multiple* Execute queries*, where the number of participants *n* and period *j* are selected by *A*. The real execution of Ψ is given by

(4) $$\begin{array}{c} param=\left\{\begin{array}{c} \left(G_{1},G_{2},e\right)⟵\text{PKG};P⟵G_{1};s⟵{Z_{q}}^{*};P_{pub}=s\cdot P;\\ QID_{1},\ldots ,QID_{n},RID_{1,j},\ldots ,RID_{n,j}⟵G_{1};\\ DID_{1,j}=\left(QID_{1}+RID_{1,j}\right)\cdot s,\ldots ,DID_{n,j}=\left(QID_{n}+RID_{n,j}\right)\cdot s:\\ \left(G_{1},G_{2},e,P,P_{pub},PID\right) \end{array}\right\},\\ Real=\left\{\begin{array}{c} a_{1},\ldots ,a_{n},h_{1},\ldots ,h_{n},r_{1},\ldots ,r_{n},w_{1},\ldots ,w_{n}⟵{Z_{q}}^{*};\\ P_{1}=a_{1}P,\ldots ,P_{n}=a_{n}P;V_{1}=DID_{1,j}+a_{1}h_{1}P_{pub},\ldots ,V_{n}=DID_{n,j}+a_{n}h_{n}P_{pub};\\ D_{1}=e{\left(P_{2}-P_{n},P_{pub}\right)}^{a_{1}},\ldots ,D_{n}=e{\left(P_{1}-P_{n-1},P_{pub}\right)}^{a_{n}};\\ \alpha_{1}=r_{1}P,\ldots ,\alpha_{n}=r_{n}P;\beta_{1}=r_{1}\left(P_{2}-P_{n}\right),\ldots ,\beta_{n}=r_{n}\left(P_{1}-P_{n-1}\right);\\ \gamma_{1}=r_{1}P_{1}+w_{1}a_{1}P_{pub},\ldots ,\gamma_{n}=r_{n}P_{n}+w_{n}a_{n}P_{pub};\\ T=\left(P_{1},\ldots ,P_{n},V_{1},\ldots ,V_{n},D_{1},\ldots ,D_{n},\alpha_{1},\ldots ,\alpha_{n},\beta_{1},\ldots ,\beta_{n},\gamma_{1},\ldots ,\gamma_{n}\right);\\ SK_{j}=e{\left(a_{1}P_{n},P_{pub}\right)}^{n}\cdot D_{1}^{n-1}\cdot D_{2}^{n-2}\cdots D_{n-1}:\left(j,T,SK_{j}\right) \end{array}\right\}, \end{array}$$

where *T* denotes the transcript and *SK*_*j*_ is the group session key for period *j*.

In* Real*, each *D*_*i*_ = *e*(*P*_*i*+1_ − *P*_*i*−1_, *P*_*pub*_)^*a*_*i*_^ = *e*(*a*_*i*_ *P*_*i*+1_, *P*_*pub*_)/*e*(*a*_*i*_ *P*_*i*−1_, *P*_*pub*_) = *e*(*a*_*i*_ *a*_*i*+1_ *P*, *P*_*pub*_)/*e*(*a*_*i*−1_ *a*_*i*_ *P*, *P*_*pub*_). by the bilinear pairing operations. We can use a random value *d*_1,2_ ∈ *Z*_q_* to substitute *a*_1_ · *a*_2_. Thus, a new distribution *Fake*_1_ is obtained as follows:

(5) $$\begin{array}{c} Fake_{1}=\left\{\begin{array}{c} d_{1,2},a_{1},\ldots ,a_{n},h_{1},\ldots ,h_{n},r_{1},\ldots ,r_{n},w_{1},\ldots ,w_{n}⟵{Z_{q}}^{*};\\ P_{1}=a_{1}P,\ldots ,P_{n}=a_{n}P;\\ V_{1}=DID_{1,j}+a_{1}h_{1}P_{pub},\ldots ,V_{n}=DID_{n,j}+a_{n}h_{n}P_{pub};\\ D_{1}=\frac{e\left(d_{1,2}P,P_{pub}\right)}{e\left(a_{n}a_{1}P,P_{pub}\right)},D_{2}=\frac{e\left(a_{2}a_{3}P,P_{pub}\right)}{e\left(d_{1,2}P,P_{pub}\right)},\ldots ,D_{n}=\frac{e\left(a_{n}a_{1}P,P_{pub}\right)}{e\left(a_{n-1}a_{n}P,P_{pub}\right)};\\ \alpha_{1}=r_{1}P,\ldots ,\alpha_{n}=r_{n}P;\beta_{1}=r_{1}\left(P_{2}-P_{n}\right),\ldots ,\beta_{n}=r_{n}\left(P_{1}-P_{n-1}\right);\\ \gamma_{1}=r_{1}P_{1}+w_{1}a_{1}P_{pub},\ldots ,\gamma_{n}=r_{n}P_{n}+w_{n}a_{n}P_{pub};\\ T=\left(P_{1},\ldots ,P_{n},V_{1},\ldots ,V_{n},D_{1},\ldots ,D_{n},\alpha_{1},\ldots ,\alpha_{n},\beta_{1},\ldots ,\beta_{n},\gamma_{1},\ldots ,\gamma_{n}\right);\\ SK_{j}=e{\left(a_{1}P_{n},P_{pub}\right)}^{n}\cdot D_{1}^{n-1}\cdot D_{2}^{n-2}\cdots D_{n-1}:\left(j,T,SK_{j}\right) \end{array}\right\}. \end{array}$$

Note that *A* can obtain all private keys *D* *ID*_*i*,*j*_ and hash values *h*_*i*_ by making* Corrupt* and* Hash queries*. It means that *A* can compute all *a*_*i*_ · *P*_*pub*_ = *h*_*i*_^−1^ · (*V*_*i*_ − *D* *ID*_*i*,*j*_) for *i* = 1, 2,…, *n*. Since the discrete logarithm assumption in *G*_1_ is intractable, *A* cannot obtain some information about *a*_*i*_ from *a*_*i*_ · *P*_*pub*_ for *i* = 1, 2,…, *n*.

In the following claim, we want to show that to distinguish two distributions* Real* from *Fake*_1_ can be reduced to solve the decisional bilinear Diffie-Hellman (DBDH) problem. Let *ε*(*t*) = *Adv*_*DBDH*_(*t*).

*Claim*. For any algorithm *A* with running time *t*, we have

(6) |Pr[(j,T,SKj)⟵Real|A(j,T,SKj)=1]   −Pr[(j,T,SKj)⟵Fake1|A(j,T,SKj)=1]|  ≤ε(t).

*Proof*. As mentioned above, each *D*_*i*_ = *e*(*a*_*i*_ *a*_*i*+1_ *P*, *P*_*pub*_)/*e*(*a*_*i*−1_ *a*_*i*_ *P*, *P*_*pub*_) = *e*(*P*, *P*_*pub*_)^*a*_*i*_*a*_*i*+1_^ *e*(*P*,*P*_*pub*_)^*a*_*i*−1_*a*_*i*_^. Here, we use Γ_*i*,*i*+1_ to substitute *e*(*P*, *P*_*pub*_)^*a*_*i*_*a*_*i*+1_^ and then each *D*_*i*_ can be written into Γ_*i*,*i*+1_/Γ_*i*−1,*i*_ for *i* = 1, 2,…, *n*. Hence, the group session key *SK*_*j*_ also can be written into (Γ_*n*,1_)^*n*^ · *D*_1_^*n*−1^ · *D*_2_^*n*−2^ ⋯ *D*_*n*−1_, where (Γ_*n*,1_)^*n*^ = *e*(*P*, *P*_*pub*_)^*na*_*n*_*a*_1_^ = *e*(*a*_1_ *P*_*n*_,*P*_*pub*_)^*n*^.

To solve the DBDH problem, we use a technique to dispose the related parameter. Considering the following algorithm *D* which inputs *P*_*a*_ = *aP*,*P*_*b*_ = *bP*, and *P*_*c*_ = *cP* ∈ *G*_1_ for some *a*, *b*, *c*∈_*R*_ *Z*_q_*. *D* first generates (*j*, *T*, *SK*_*j*_) according to the distribution *Dist* ^1^. Then, *D* runs *A*(*j*, *T*, *SK*_*j*_) and outputs whatever *A* outputs. The distribution *Dist* ^1^ is defined as follows:

(7) $$\begin{array}{c} {Dist}^{1}=\left\{\begin{array}{c} a_{1},\ldots ,a_{n},h_{1},\ldots ,h_{n},u_{1},\ldots ,u_{n-2},r_{1},\ldots ,r_{n},w_{1},\ldots ,w_{n}⟵{Z_{q}}^{*};\\ P_{1}=a_{1}P,\ldots ,P_{n}=a_{n}P;V_{1}=DID_{1,j}+a_{1}h_{1}P_{pub},\ldots ,V_{n}=DID_{n,j}+a_{n}h_{n}P_{pub};\\ \Gamma_{1,2}=g_{sab}\in G_{2},\Gamma_{2,3}=e{\left(P_{b},P_{pub}\right)}^{u_{1}},\Gamma_{i,i+1}=e{\left(P,P_{pub}\right)}^{u_{i-2}u_{i-1}}\;\text{for}\;i=3\;\text{to}\;n-1\\ \Gamma_{n,1}=e{\left(P_{a},P_{pub}\right)}^{u_{n-2}};D_{1}=\frac{\Gamma_{1,2}}{\Gamma_{n,1}},\ldots ,D_{n}=\frac{\Gamma_{n,1}}{\Gamma_{n-1,n}};\\ \alpha_{1}=r_{1}P,\ldots ,\alpha_{n}=r_{n}P;\beta_{1}=r_{1}\left(P_{2}-P_{n}\right),\ldots ,\beta_{n}=r_{n}\left(P_{1}-P_{n-1}\right);\\ \gamma_{1}=r_{1}P_{1}+w_{1}a_{1}P_{pub},\ldots ,\gamma_{n}=r_{n}P_{n}+w_{n}a_{n}P_{pub};\\ T=\left(P_{1},\ldots ,P_{n},V_{1},\ldots ,V_{n},D_{1},\ldots ,D_{n},\alpha_{1},\ldots ,\alpha_{n},\beta_{1},\ldots ,\beta_{n},\gamma_{1},\ldots ,\gamma_{n}\right);\\ SK_{j}={\left(\Gamma_{n,1}\right)}^{n}\cdot D_{1}^{n-1}\cdot D_{2}^{n-2}\cdots D_{n-1}:\left(j,T,SK_{j}\right) \end{array}\right\}. \end{array}$$

Note that this distribution depends on *P*_*a*_, *P*_*b*_, and *P*_*c*_.

By the above distribution *Dist* ^1^, let Γ_1,2_ = *e*(*P*,*P*_*pub*_)^*ab*^ = *e*(*P*,*P*)^*sab*^. Then, we can obtain another distribution called *Dist*_*DBDH*_^1^. Obviously, *Dist*_*DBDH*_^1^ is identical to* Real* because

(8) SKj  =(Γn,1)(Γ1,2)(Γ2,3)⋯(Γn−2,n−1)(Γn−1,n)=e(Pa,Ppub)un−2·e(P,P)sab·e(Pb,Ppub)u1⋯e(P,Ppub)un−4un−3  ·e(P,Ppub)un−3un−2  =e(P,P)sun−2a+sab+sbu1+⋯+sun−4un−3+sun−3un−2.  

Similarly, let Γ_1,2_ = *e*(*P*_*c*_, *P*_*pub*_) = *e*(*P*,*P*)^*sc*^ for some *c* ≠ *ab* ∈ *Z*_q_*. Then, we can obtain another distribution called *Dist*_*Random*_^1^. Obviously, *Dist*_*Random*_^1^ is identical to *Fake*_1_ because

(9) SKj  =  (Γn,1)(Γ1,2)(Γ2,3)⋯(Γn−2,n−1)(Γn−1,n)=e(Pa,Ppub)un−2·e(P,P)sc·e(Pb,Ppub)u1⋯e(P,Ppub)un−4un−3·e(P,Ppub)un−3un−2=e(P,P)sun−2a+sc+sbu1+⋯+sun−4un−3+sun−3un−2.

Therefore, we have

(10) |Pr[(j,T,SKj)⟵Real|A(j,T,SKj)=1]   −Pr[(j,T,SKj)⟵Fake1|A(j,T,SKj)=1]|  ≤ε(t).

This completes the proof of claim.

Using the same process in *Fake*_1_, we can define other distributions *Fake*_*i*_ for *i* = 2, 3,…, *n*. By a similar approach in claim, we can obtain the following n-1 equations in (11) for any adversary *A* with running time *t*

(11) |Pr[(j,T,SKj)⟵Fake1|A(j,T,SKj)=1]   −Pr[(j,T,SKj)⟵Fake2|A(j,T,SKj)=1]|  ≤ε(t),⋮|Pr[(j,T,SKj)⟵Faken−1|A(j,T,SKj)=1]   −Pr[(j,T,SKj)⟵Faken|A(j,T,SKj)=1]|  ≤ε(t).

This implies

(12) |Pr[(j,T,SKj)⟵Real|A(j,T,SKj)=1]   −Pr[(j,T,SKj)⟵Faken|A(j,T,SKj)=1]|  ≤n·ε(t).

In *Fake*_*n*_, the values *d*_1,2_, *d*_2,3_,…, *d*_*n*−1,*n*_, *d*_*n*,1_ are constrained by *T* according to the following *n* equations:

(13) log⁡g⁡D1  =  s·(d1,2  −  dn,1),log⁡g⁡D2=  s·(d2,3−d1,2),…,log⁡g⁡Dn=  s·(dn,1−dn−1,n),

where *g* = *e*(*P*, *P*). Since *SK*_*j*_ can be expressed as *e*(*P*, *P*)^*sd*_1,2_+*sd*_2,3_+⋯+*sd*_*n*,1_^, we can obtain log⁡_*g*_ *SK*_*j*_ = *sd*_1,2_ + *sd*_2,3_ + ⋯+*sd*_*n*,1_. Because *sd*_1,2_ + *sd*_2,3_ + ⋯+*sd*_*n*,1_ is linear and independent from the set {log⁡_*g*_ *D*_*i*_ = *s* · (*d*_*i*,*i*+1_ − *d*_*i*−1,*i*_) | *i* = 1, 2,…, *n*}, it implies that *SK*_*j*_ is independent for the transcript *T*. In other words, for any adversary *A*

(14) Pr[(j,T,SKj,0)⟵Faken,SKj,1⟵G2 ∣ A(j,T,SKj,b)   =1,b⟵{0,1}]=12.

Therefore, the advantage of *A* on the event ¬*Forge* is bounded by 2*n* · *Adv*_*DBDH*_(*t*). Combining the two cases, the advantage of *A* is bounded by

(15) $$\begin{array}{c} Adv_{\Psi }^{RIDAGKE\text{-}fs}\left(t,1,q_{s}\right)\leq 2n\cdot Adv_{DBDH}\left(t\right)+Adv_{\Psi }^{forge}\left(t\right). \end{array}$$

Finally, a standard hybrid argument immediately demonstrates that

(16) AdvΨRIDAGKE-fs(t,qex,qs)≤  2nqex·AdvDBDH(t)  +AdvΨforge(t)  for  qex  >1.

Under the decisional bilinear Diffie-Hellman (DBDH) assumption, the advantage *Adv*_*DBDH*_(*t*) is negligible. By Theorem 4, the advantage *Adv*_Ψ_^*forge*^(*t*) is also negligible. Hence, we can obtain that the advantage *Adv*_Ψ_^*RIDAGKE*-*fs*^(*t*, *q*_*ex*_, *q*_*s*_) is negligible according to the result in Theorem 5. It implies that the proposed RID-AGKE protocol Ψ is a secure RID-AGKE providing forward secrecy.

*Identifying Malicious Participant*

**Theorem 6**

The proposed RID-AGKE protocol can identify malicious participants.

**Proof**

Note that in Round 2 a noninteractive confirmed computation technique is involved in adopted pairing-based signature scheme. The security of confirmed computation had been proven in [6]. Concretely, each participant *U*_*i*_ can confirm the broadcasted value *D*_*k*_ is computed by *U*_*k*_ using her/his secret *a*_*k*_ after passing two verifying equations for *k* = 1, 2,…, *n* except *i*. Hence, if there is a participant *U*_*m*_ who broadcasts a wrong *D*_*m*_ to disturb the group session key establishing, he will be identified as a malicious participant. In other words, the proposed RID-AGKE protocol can identify malicious participants by using the confirmed computation technique.

## 6. Performance Analysis and Comparisons

For convenience to evaluate the computational cost, we focus on the time-consuming pairing-based operations as follows:

- *TG*_*e*_: the time of executing a bilinear map operation *e* : *G*_1_ × *G*_1_ → *G*_2_;
- *TG*_*mul*_: the time of executing a point scalar multiplication operation in *G*_1_;
- *TG*_*H*_: the time of executing a map-to-point hash function *H*_1_, *H*_2_ : {0,1}* → *G*_1_;
- *T*_*exp*_: the time of executing a modular exponentiation operation over a finite field *F*_*p*_, where *p* is a large prime number;
- *T*_*inv*_: the time of executing a modular multiplicative inverse operation over a finite field *F*_*p*_, where *p* is a large prime number.

Here, we first analyze the computational cost of our protocol. In Round 1, 2*TG*_*mul*_ is required to compute (*P*_*i*_, *V*_*i*_). In Round 2, each participant requires 3*TG*_*e*_ + 7*TG*_*mul*_ + 4*TG*_*H*_ to verify (*ID*_*i*+*k*_, *j*, *P*_*i*+*k*_, *V*_*i*+*k*_) for *k* ∈ {−1, 1} and to generate (*D*_*i*_, *α*_*i*_, *β*_*i*_, *γ*_*i*_). In the group key computation phase, (3*n* − 1)*TG*_*e*_ + *n* *TG*_*mul*_ + (*n* − 1)*T*_*exp*_ is required to verify all (*ID*_*i*_, *j*, *D*_*i*_, *α*_*i*_, *β*_*i*_, *γ*_*i*_) and to compute a group key *SK*_*j*_. Note that to evaluate *SK*_*j*_ = *e*(*a*_*i*_·*P*_*i*−1_,*P*_*pub*_)^*n*^ · *D*_*i*_^*n*−1^ · *D*_*i*+1_^*n*−2^ · ··*D*_*i*−2_ is required *TG*_*e*_ + *TG*_*mul*_ since *SK*_*j*_ = *A*_*i*−1_ · *A*_*i*_ · *A*_*i*+1_ ⋯ *A*_*i*−2_, where *A*_*i*−1_ = *e*(*a*_*i*_ · *P*_*i*−1_, *P*_*pub*_), *A*_*i*_ = *A*_*i*−1_ · *D*_*i*_, *A*_*i*+1_ = *A*_*i*_ · *D*_*i*+1_,…, and *A*_*i*−2_ = *A*_*i*−3_ · *D*_*i*−2_. As a result, each participant requires (3*n* + 2)*TG*_*e*_ + (*n* + 9)*TG*_*mul*_ + 4*TG*_*H*_ + (*n* − 1)*T*_*exp*_ in our protocol.

In Table 1, we compare our RID-AGKE protocol with four previously proposed AGKE protocols which include Tseng's AGKE protocol [25], Choi et al.'s ID-AGKE protocol [26], Wu et al.'s ID-AGKE protocol [6], and Wu et al.'s RID-AGKE protocol [4] in terms of the public key setting, number of rounds, computational cost, and security properties. One recent non-ID-based and non-RID-based AGKE protocol with identifying malicious participants was proposed by Tseng [25]. Since Tseng's protocol is based on the ElGmal system [29], each participant must verify the other participants' certificates for participant authentication. It will increase the required computational costs for verifying certificates, besides (8*n* − 2)*T*_*exp*_ + (*n* + 1)*T*_*inv*_. On the contrary, Choi et al.'s ID-AGKE [26], Wu et al.'s ID-AGKE [6], Wu et al.'s RID-AGKE [4], and our protocol rely on the IDPKS system [8] or the R-IDPKS system [14]. Thus, they need not manage and verify the participants' certificates. However, Choi et al.'s ID-AGKE [26] suffered from an insider colluding attack demonstrated by Wu and Tseng [27].

For Wu et al.'s ID-AGKE [6], Wu et al.'s RID-AGKE [4], and our protocol, they are provably secure and are able to resist malicious participants. It is easy to see that our protocol is more efficient than Wu et al.'s ID-AGKE [6] even though both protocols can identify malicious participants via confirmed computation approach. More importantly, Wu et al.'s ID-AGKE protocol [6] does not provide a solution to revoke the compromised or misbehaving user in the group. It is very serious because these revoked participants should not be allowed to establish a common key with other legal (nonrevoked) participants. In another aspect, Wu et al.'s RID-AGKE [4] is a three-round protocol and adopts explicit key confirmation approach to resist malicious participants. Though their protocol can detect the existence of malicious participants, it cannot still identify malicious participant. Our proposed RID-AGKE is a two-round protocol and provides an active detection mechanism to identify malicious participants. According to Table 1, the advantage of our protocol is demonstrated.

## 7. Conclusions

In this paper, we have fused the Tseng-Tsai R-IDPKS system and a noninteractive confirmed computation technique to propose the first RID-AGKE protocol with identifying malicious participants. The framework and security notions for RID-AGKE protocols have been defined to formalize the possible threats and attacks. When compared with the recently proposed ID/RID-AGKE protocols resistant to malicious participants, our protocol has better performance and provides an active detection way to identify malicious participants. In the random oracle model and under two mathematical assumptions (CDH and DBDH), we have proven that the proposed protocol is a secure RID-AGKE protocol with forward secrecy and identifying malicious participants.

## Figures and Tables

**Table 1:** Comparisons between our protocol and the previously proposed AGKE protocols.

	Tseng's AGKE [25]	Choi et al.'s ID-AGKE [26]	Wu et al.'s ID-AGKE [6]	Wu et al.'s RID-AGKE [4]	Our protocol
Public key setting	ElGmal	IDPKS	IDPKS	R-IDPKS	R-IDPKS

Certificate management	Required	Not required	Not required	Not required	Not required

Rounds	2	2	2	3	2

Computational cost for each participant	(8*n* − 2)*T*_*exp*_ + (*n* + 1)*T*_*inv*_	6*TG*_*e*_ + (*n* + 11)*TG*_*mul*_ + (*n* + 3)*TG*_*H*_	(3*n* + 3)*TG*_*e*_ + (*n* + 10)*TG*_*mul*_ + 3*TG*_*H*_ + (*n* − 1)*T*_*exp*_	8*TG*_*e*_ + (2*n* + 8)*TG*_*mul*_ + 4*n* *TG*_*H*_	(3*n* + 2)*TG*_*e*_ + (*n* + 9)*TG*_*mul*_ + 4*TG*_*H*_ + (*n* − 1)*T*_*exp*_

Security	Provably secure	Existing attack [27]	Provably secure	Provably secure	Provably secure

Revocation functionality	Using CRL [28]	No	No	Yes	Yes

Resistant to malicious participants	Yes (confirmed computation)	No	Yes (confirmed computation)	Yes (explicit key confirmation)	Yes (confirmed computation)

Identifying malicious participants	Yes	No	Yes	No	Yes

## Notations

*e*: A bilinear map, *e* : *G*_1_ × *G*_1_ → *G*_2_, defined in Section 2.1
*s*: The system private key, *s* ∈ *Z*_*q*_*
*P*: A generator of group *G*_1_
*P*_*pu**b*_: The system public key, *P*_*pub*_ = *s* · *P*
*ID*_*i*_: The identity of participant *U*_*i*_
*D**ID*_*i*_: The participant *U*_*i*_'s initial private key
*T**ID*_*i*,*j*_: The participant *U*_*i*_'s update key for period *j*
*D**ID*_*i*,*j*_: The participant *U*_*i*_'s private key for period *j*, *D* *ID*_*i*,*j*_ = *D* *ID*_*i*_ + *T* *ID*_*i*,*j*_
*H*_1_: A map-to-point hash function, *H*_1_ : {0,1}* → *G*_1_
*H*_2_: A map-to-point hash function, *H*_2_ : {0,1}* → *G*_1_
*H*_3_: A hash function, *H*_3_ : {0,1}* × *G*_1_ → *Z*_*q*_
*H*_4_: A hash function, *H*_4_ : {0,1}* × *G*_1_^3^ → *Z*_*q*_.
